# Isolation of cancer cells with augmented spheroid-forming capability using a novel tool equipped with removable filter

**DOI:** 10.18632/oncotarget.26092

**Published:** 2018-09-21

**Authors:** Emi Fujibayashi, Norikazu Yabuta, Yukihiro Nishikawa, Toshihiro Uchihashi, Daisaku Miura, Kyoko Kurioka, Susumu Tanaka, Mikihiko Kogo, Hiroshi Nojima

**Affiliations:** ^1^ First Department of Oral and Maxillofacial Surgery, Graduate School of Dentistry, Osaka University, Suita 565-0871, Osaka, Japan; ^2^ Department of Molecular Genetics, Research Institute for Microbial Diseases, Osaka University, Suita 565-0871, Osaka, Japan; ^3^ Department of Pharmacy, Hyogo University of Health Sciences, Kobe 650-8530, Japan

**Keywords:** spheroid, HHAT, SAS, tumor, invasion

## Abstract

Three-dimensional (3D) cell culture systems have been used to obtain multicellular spheroidal cell aggregates, or spheroids, from cancer cells. However, it is difficult to efficiently prepare large tumor-derived spheroids from cancer cells. To circumvent this problem, we here used a tool equipped with removal membrane, called Spheroid Catch, for the selection and enrichment of large-sized and/or size-matched spheroids from human squamous cell carcinoma (SAS cells) without loss of recovery. After a five-round process of selection and enrichment, we successfully isolated a subpopulation of SAS cells with augmented spheroid-forming capability, named eSAS: the efficiency of spheroid formation is 28.5% (eSAS) vs 16.8% (parental SAS). Notably, we found that some of eSAS cells survived after exposure of high doses of cisplatin in 3D culture. Moreover, orthotopic implantation by injecting eSAS cells into the tongues of nude mice showed reduced survival rate and increased tumor growth compared with those of nude mice injected with SAS cells. These results suggest that spheroids exhibiting properties of higher spheroid forming capacity can be efficiently collected by using Spheroid Catch. Indeed, genome-wide cDNA microarray and western blot analyses demonstrated higher mRNA and protein levels of hedgehog acyltransferase (HHAT), which is associated with stem maintenance in cell carcinoma by catalysing the N-palmitoylation of Hedgehog proteins, in eSAS cells than in SAS cells. We propose that Spheroid Catch could be useful for the study of spheroids, and potentially organoids, in the basic and clinical sciences, as an alternative method to other type of cell strainers.

## INTRODUCTION

The formation of multicellular spheroidal cell aggregates, or spheroids, is a conspicuous characteristic of cancer stem cells (CSCs) and tumor-initiating cells (TICs) that possess the ability for self-renewal, proliferation and generation of downstream progenitor cells to promote tumor growth [1]. Since CSCs and TICs within the tumor mass have been proposed to mediate chemoresistance and cancer recurrence [2, 3], the development of an efficient experimental system to study the molecular mechanisms of tumor-derived spheroids in cultured cancer cells would facilitate considerably the study of the mechanisms involved in chemoresistance and cancer recurrence. Recent advances in three-dimensional (3D) cell culture systems that mimic *in vivo* physiology allow observation of spheroid formation by a variety of cancer cell lines [4–7]. 3D culture is also used for efficient antitumor drug screening to exclude false-positive compounds from entry into clinical trials [8]. However, for many cancer cell lines, the efficiency of spheroid formation is low and/or the size of the spheroids is small, which hampers detailed investigation of the molecular mechanisms of spheroids *in vivo* [1]. Moreover, the production of spheroids with different sizes and shapes may influence drug efficacy and toxicity, leading to high dropout rates, and the loss of time and financial resources [8]. Thus, the development of a convenient and simple technique that allows selection of large-sized and/or size-matched spheroids in targeted cancer cell lines is under active investigation.

We previously invented a simple and convenient leukocyte trapping apparatus, termed LeukoCatch^TM^. The device, which was equipped with a Leuko-filter at the bottom of a syringe-shaped container, was successfully used to prepare a total cell extract of white blood cells from cancer patients and healthy volunteers within minutes [9, 10]. We also manufactured another simple and efficient method, Leuko-elute, equipped with a Leuko-filter at the bottom of a cup-shaped container. Leuko-elute can be used for the preparation of live leukocytes from peripheral blood [11], which is valuable at the bedside because live leukocytes can be obtained from patients within just a few minutes. Leuko-elute is more useful than other commercially-available tools, such as cell strainer (Corning Co. Ltd.), because the bottom of the container can be readily detached with forceps in the tissue culture medium, unlike the undetachable cell strainer. We proposed that Leuko-elute could be used to develop a novel tool to trap large-sized and/or size-matched spheroids if the Leuko-filter was replaced by mesh of variable size.

In the present study, we used an easy-to-use and low-cost novel tool, called Spheroid Catch, which is a tapered polypropylene cylinder with six spokes at the bottom to support the removable mesh, for the selection of large-sized and/or size-matched spheroids. We tested the efficiency of Spheroid Catch for the isolation of very large spheroids using a human tongue squamous cell carcinoma cell line, SAS, because this cell line forms comparatively larger spheroids in 3D cell culture systems [12–15] than other cell lines, such as prostate cancer [13, 16–18] and colorectal cancer cell lines [4]. Based on the results obtained here, we propose that Spheroid Catch has potential as a new 3D cell culture system for the study of spheroids.

## RESULTS

### Preparation and usage of spheroid catch

**Figure 1:**
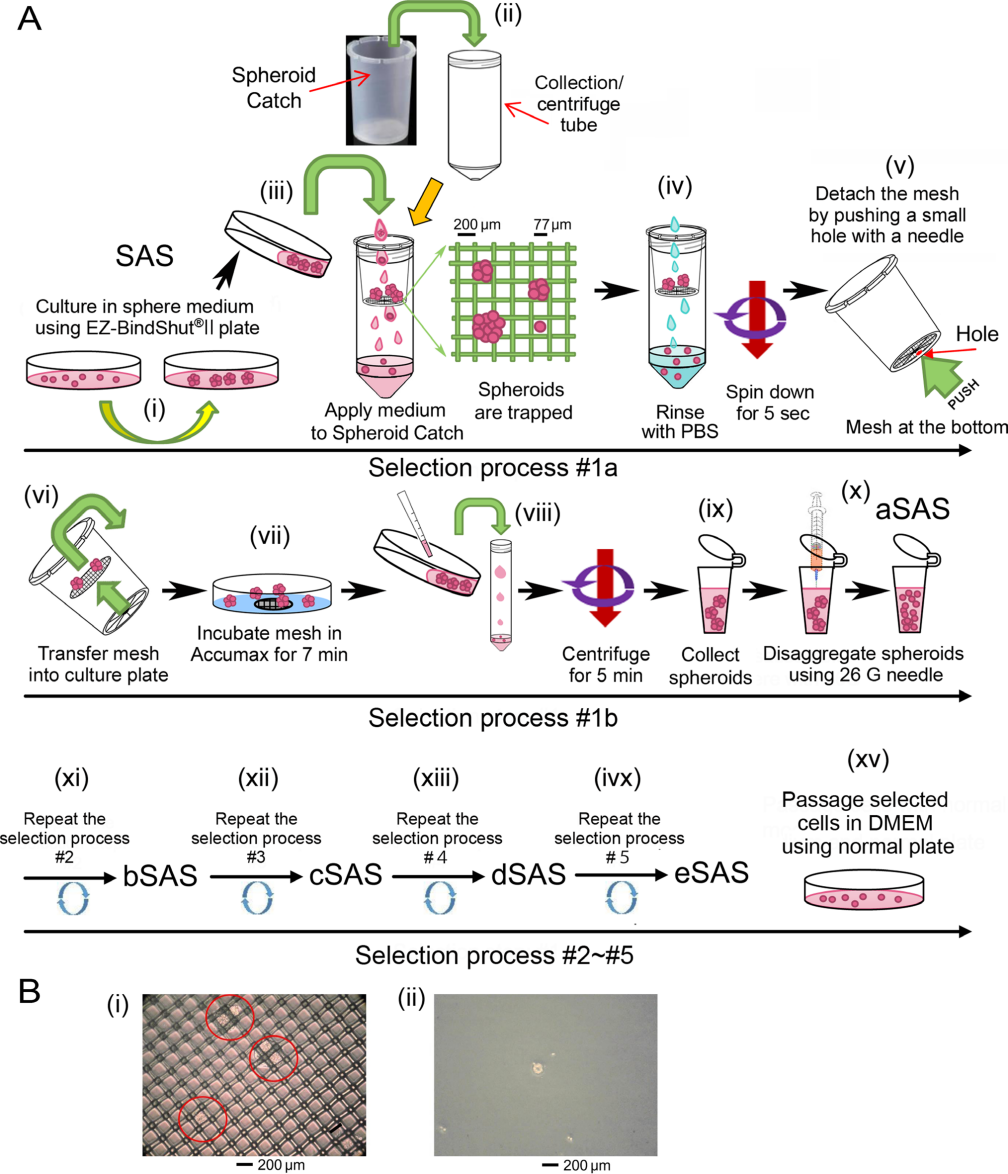
Typical manipulation of Spheroid Catch (**A**) Schematic drawings for the Spheroid Catch protocol described in detail in the Results section. aSAS, bSAS, cSAS, dSAS, and eSAS denote the selected SAS cells after the first, second, third, fourth, and fifth selection rounds, respectively. (**B**) Typical microscopic images for the spheroids trapped by Spheroid Catch are highlighted by red circles (i) and an image of the selected spheroid incubated under tissue culture conditions (ii) is shown.

SAS cells cultured in spheroid-forming medium (SFM) on a spheroid-forming plate (SFP) were collected and transferred to Spheroid Catch inserted into a collection tube (Figure 1A-i, -ii and -iii). Under gravity filtration, spheroids larger than 77 μm were trapped by the mesh. After rinsing the mesh with phosphate buffered saline without calcium or magnesium (PBS(−)), the small spheroids that stuck to the mesh were removed by centrifugation (Figure 1A-iv). Next, the mesh at the bottom was detached by pushing a small hole with a needle or a tip of forceps (Figure 1A-v, vi), and the mesh was transferred into a culture plate containing 1 mL Accumax to enzymatically detach the trapped spheroids by incubation for 7 min (Figure 1A-vii). Then, spheroids were collected by centrifugation (Figure 1A-viii), followed by disaggregation process using a 26 G needle (Figure 1A-ix, x). This selection process (#1a) was repeated until many large-sized spheroids were obtained (Figure 1A-xi~xv). A typical image of a mesh harboring large-sized spheroids (Figure 1B-i) that were recovered in fresh SFM (Figure 1B-ii) is presented.

### Selection of SAS cells with increased spheroid formation ability using Spheroid Catch

**Figure 2:**
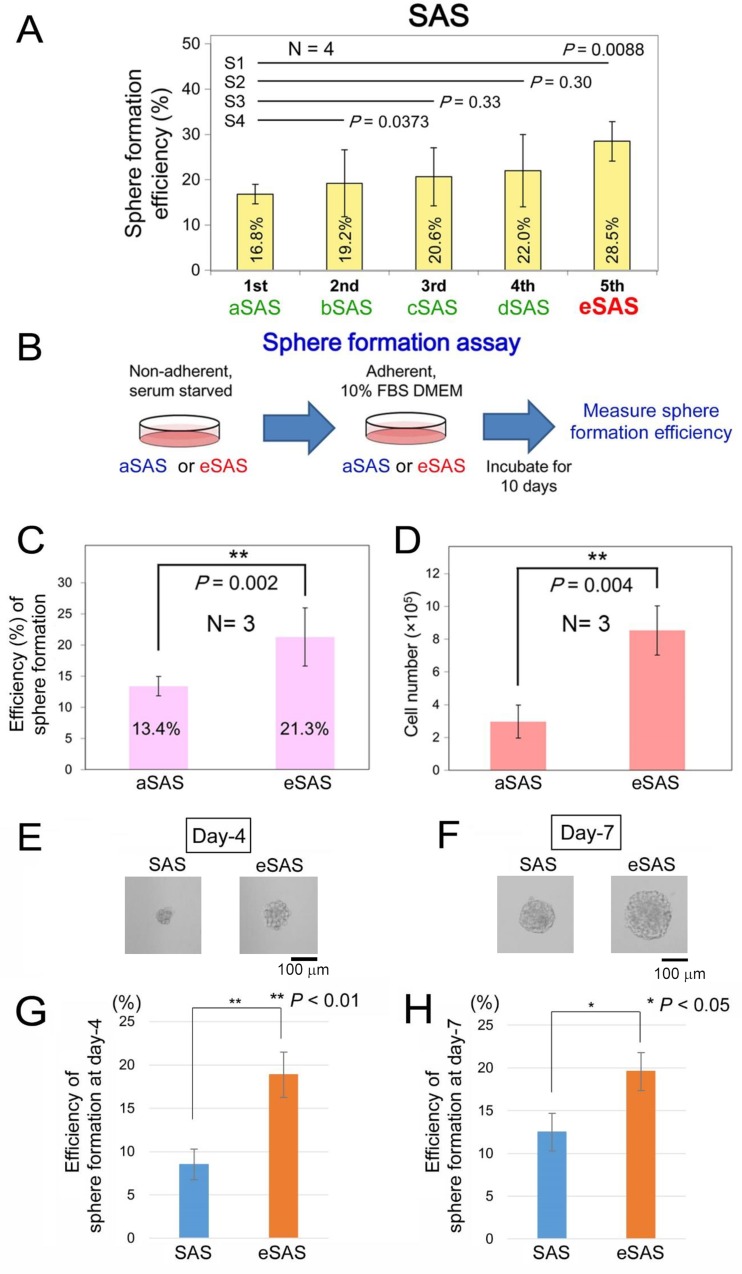
Selection of aSAS ~ eSAS cell lines using Spheroid Catch (**A**) Bar graphs indicate the spheroid formation efficiencies measured in SFM using SFPs for aSAS, bSAS, cSAS, dSAS and eSAS cells after the first, second, third, fourth and fifth selection rounds, respectively. (**B**) Schematic drawings of the spheroid formation assay protocol for aSAS and eSAS cells obtained using Spheroid Catch. (**C**, **D**) Bar graphs indicate the spheroid formation efficiencies (C) and cell number (D) measured in SFM using SFPs for the parental SAS and eSAS cells after incubation for 10 days in standard culture medium. (**E**, **F**) Typical image of SAS and eSAS spheroids, isolated as single colonies from a 96-well SFP, at day 4 (E) and day 7 (F). Scale bars, 100 μm. (**G**, **H**) Bar graphs indicate the percentages of spheroid formation for the parental SAS and eSAS cells at day 4 (G) and day 7 (H). Bar graphs were constructed based on results of three independent experiments.

We previously compared the spheroid formation efficiencies of a normal cell line (TIG-1) and four cancer cell lines (LNCaP, PC-3, DU-145, and SAS) and found that SAS cells formed comparatively larger spheroids in a 3D cell culture system than other cancer cells [13]. Thus, we used this cell line to demonstrate the spheroid retention properties of Spheroid Catch. After SAS cells were subjected to Spheroid Catch five times, the percentages of cells that formed spheroids were 16.8% (aSAS), 19.2% (bSAS), 20.6% (cSAS), 22.0% (dSAS), and 28.5% (eSAS) after the first, second, third, fourth, and fifth rounds of selection, respectively (Figure 2A). These results indicate that a 1.70-fold increase (28.5% vs. 16.8%) in spheroid formation efficiency was achieved after only four selection processes.

Next, to examine whether spheroid formation efficiency (%) is retained after selection, we performed a spheroid formation assay in which aSAS or eSAS cells cultured in SFM on a SFP (non-adherent 3D plate) were transferred to standard culture medium in an adherent plate and incubated for 10 days (Figure 2B). This assay is essential to determine whether the increased spheroid-forming ability is a stably-acquired characteristic or a transient phenotype. Indeed, the spheroid formation efficiency of the eSAS cells (21.3% remained 1.59-fold higher than that of the aSAS cells (13.4%) even after a 10-day incubation period in standard culture medium on the adherent plate (Figure 2C). We also measured the number of cells in the non-adherent 3D plate and found that eSAS spheroids contained more cells than aSAS spheroids (Figure 2D), suggesting that a larger proportion of eSAS cells were prone to form spheroids than aSAS cells.

Next, we attempted to obtain colonies derived from a single SAS and eSAS cells by seeding about ten cells obtained via a step-wise dilution procedure into each well of a 96-well SFP and incubating them for up to one week in SFM. A typical image of SAS and eSAS spheroids derived from single colonies at day 4 (Figure 2E) and day 7 (Figure 2F) indicated that the eSAS cells formed larger spheroids than parental SAS cells. Bar graphs showing the percentages of spheroids at day 4, in which colonies harboring spheres with more than ten cells were defined as spheroid (Figure 2E), indicated that the acquired phenotype of increased spheroid formation efficiency is present as early as 4 days after incubation (Figure 2G). Similar bar graphs at day 7, in which colonies harboring irregular-sized globular spheres covered with a putative extracellular matrix were defined as spheroid (Figure 2F), confirmed that this phenotype was still present after 7 days incubation (Figure 2H). Based on these results, we conclude that Spheroid Catch is useful for the isolation of cells with larger-size spheroid-forming ability.

### eSAS cells exhibit increased resistance to cisplatin

**Figure 3:**
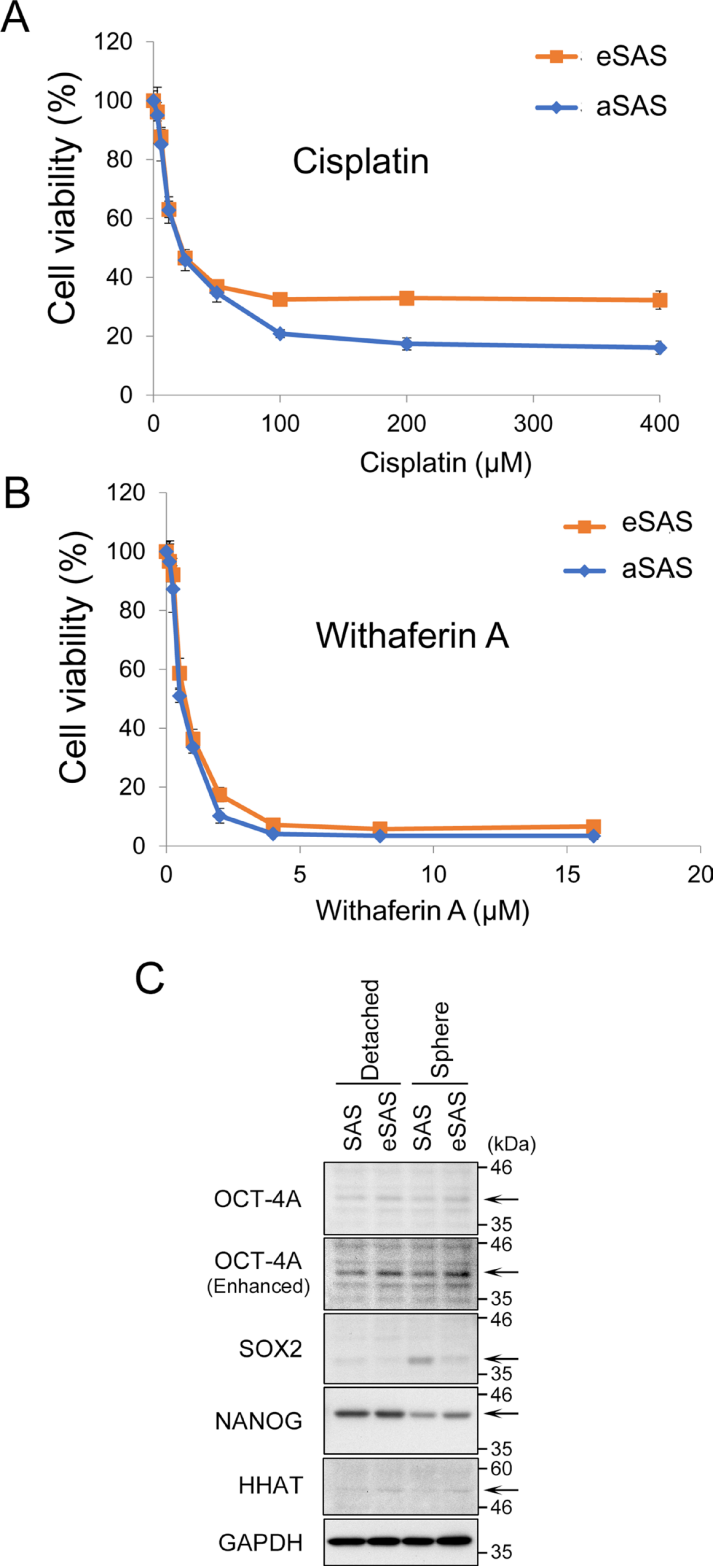
eSAS cells are more resistant to cisplatin than aSAS cells but not to WA (**A**, **B**) Line graphs indicate the cell viability (%) determined by the MTT assay after 48 hours of 3D culture in the presence of the indicated concentrations of cisplatin (A) or WA (B) for aSAS or eSAS cells. (**C**) Expression of OCT-4A, SOX2, and NANOG in SAS and eSAS cells. Wb analysis of the lysates from cells detached by trypsinization (non-spheres) and spheres growing in SFM/SFP for 4 days using the antibodies against the indicated proteins (arrows). GAPDH is a loading control. As for HHAT, also see Figure 7.

We previously demonstrated that SAS cells grown in a 3D culture are more resistant to cisplatin than SAS cells cultured in standard culture medium on adherent plates [13]. A higher level of resistance to cisplatin is a typical characteristic of cell lines with CSC-like properties [12]. Thus, we questioned whether SAS cells that showed increased spheroid formation would be more resistant to cisplatin. eSAS cells were more viable than aSAS cells when incubated in 3D culture conditions in the presence of cisplatin (Figure 3A). However, some of eSAS cells might survive after exposure of high doses of cisplatin because the IC50 is likely to be the same in eSAS and aSAS cells.

We previously demonstrated that Withaferin A (WA) kills SAS cells regardless of their spheroid formation ability, i.e., it killed SAS cells equally well under both conventional and 3D culture conditions [13]. Using 3D culture conditions, we observed that eSAS cells were as viable as aSAS cells when cultured in the presence of WA (Figure 3B). This result confirms the previous result that spheroid formation does not affect resistance to WA.

Moreover, to validate that the increased resistance of eSAS cells is due to enriched CSC-like cells, we compared the expression levels of stem cell markers, such as OCT-4A, NANOG, and SOX2, between SAS and eSAS cells. Western blotting (Wb) revealed that OCT-4A was slightly increased in eSAS cells compared with parental SAS cells under both conditions; non-spheres detached by trypsinization and spheres growing in SFM/SFP for 4 days (Figure 3C, top and second panels). The expression level of NANOG in eSAS spheres also seemed to be faintly increased compared with that in SAS spheres, both of which were lower than the levels in detached SAS and in eSAS cells (Figure 3C, fourth panel from top). Interestingly, the expression level of SOX2 was increased in the spheres of SAS cells but not eSAS cells (Figure 3C, third panel from top). These results suggest that eSAS cells acquired apparently different stem cell properties from parental SAS cells, which also suggest that it is difficult to assess the level of CSC-like cells enriched in eSAS cells by simply measuring the expression levels of typical stem cell markers.

### Tumor growth of implanted eSAS cells is increased in a mouse model of carcinoma

**Figure 4:**
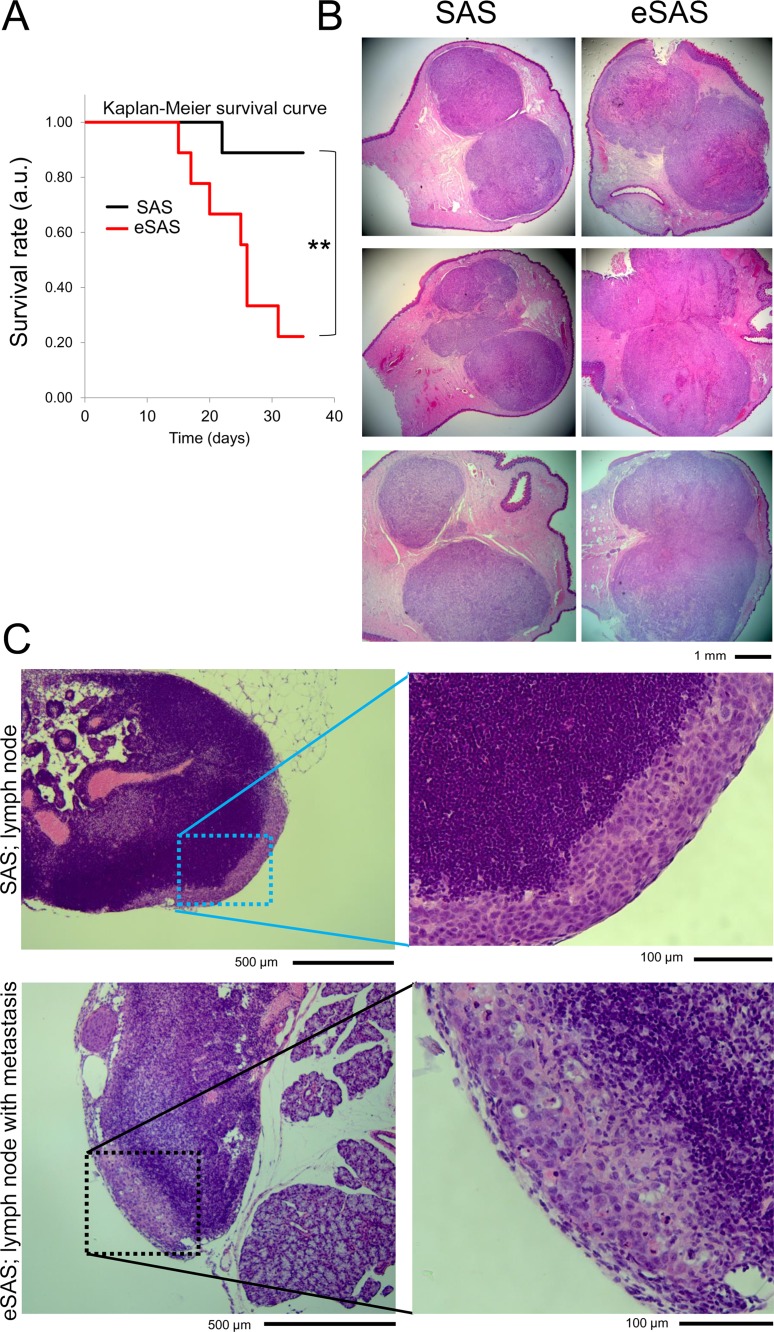
eSAS cells exhibit a reduced survival rate in nude mice (**A**) Survival curves of mice with SAS or eSAS cells implanted into the tongue. Survival rate is presented in arbitrary units (a.u.). Survival analysis of the nude mice was evaluated by the Kaplan-Meier method and the log-rank test (Peto-Peto and Cochran-Mantel-Haenszel) and generalized Wilcoxon test (Gehan-Breslow) was used to compare the data from nine SAS cell-implanted and nine eSAS cell-implanted mice. One SAS cell-implanted mouse and seven eSAS cell-implanted mice died at day 35 after implantation. ^**^P < 0.01, namely, *P* = 0.0064 as determined by Cochran-Mantel-Haenszel method. (**B**) Hematoxylin and eosin (HE) staining of dissected tongues of mice implanted with SAS (left panels) and eSAS (right panels) cells. The mice were sacrificed fourteen days after cell implantation. The six mice employed in this assay were different from those used to evaluate survival rates. Tumors were observed as regions with a dark pink color. (**C**) Typical images of cervical lymph nodes of nude mice implanted with SAS or eSAS cells after HE staining. Right panels show enlarged images of the left panels. Regions of interest are encircled by dotted squares in the left panels. The pale purple region in the right panel of mice implanted with eSAS shows the putative invasive region.

To examine whether eSAS cells display altered growth patterns in mice, we attempted orthotopic implantation by injecting parental SAS and eSAS cells into the tongues of BALB/c nude mice, because SAS and eSAS cells are derived from tongue cancers. Since it is technically difficult to measure tumor size in the mouths of mice prior to sacrifice, we first measured survival rates during the experiment, with the expectation that tumor growth *in vivo* would promote death due to eating disorders, although invasive tumor growth rather than cell growth rate was more likely to kill the animals. Indeed, the survival rate of mice injected with eSAS cells was significantly lower than that of mice injected with parental SAS cells and their time of death was earlier (Figure 4A). Second, we preformed pathological analysis of six independent mice sacrificed at fourteen days after injection; these mice were distinct from those used in survival analysis. The results showed that eSAS cells formed larger tumors than SAS cells in all of the six tested cases; dissection at their largest diameters revealed a clear boundary (Figure 4B). The larger tumor size might have been the cause of the earlier death of mice implanted with eSAS cells (Figure 4A), probably because the larger tumor size interfered with eating. Interestingly, unlike the lymph nodes of mice implanted with SAS cells, a lymph node of one of the mice implanted with eSAS cells showed putative invasive cancer cells (Figure 4C). These results suggest that eSAS cells are more malignant in mice than SAS cells (see Discussion).

### The growth of eSAS cells is slower than that of SAS cells

**Figure 5:**
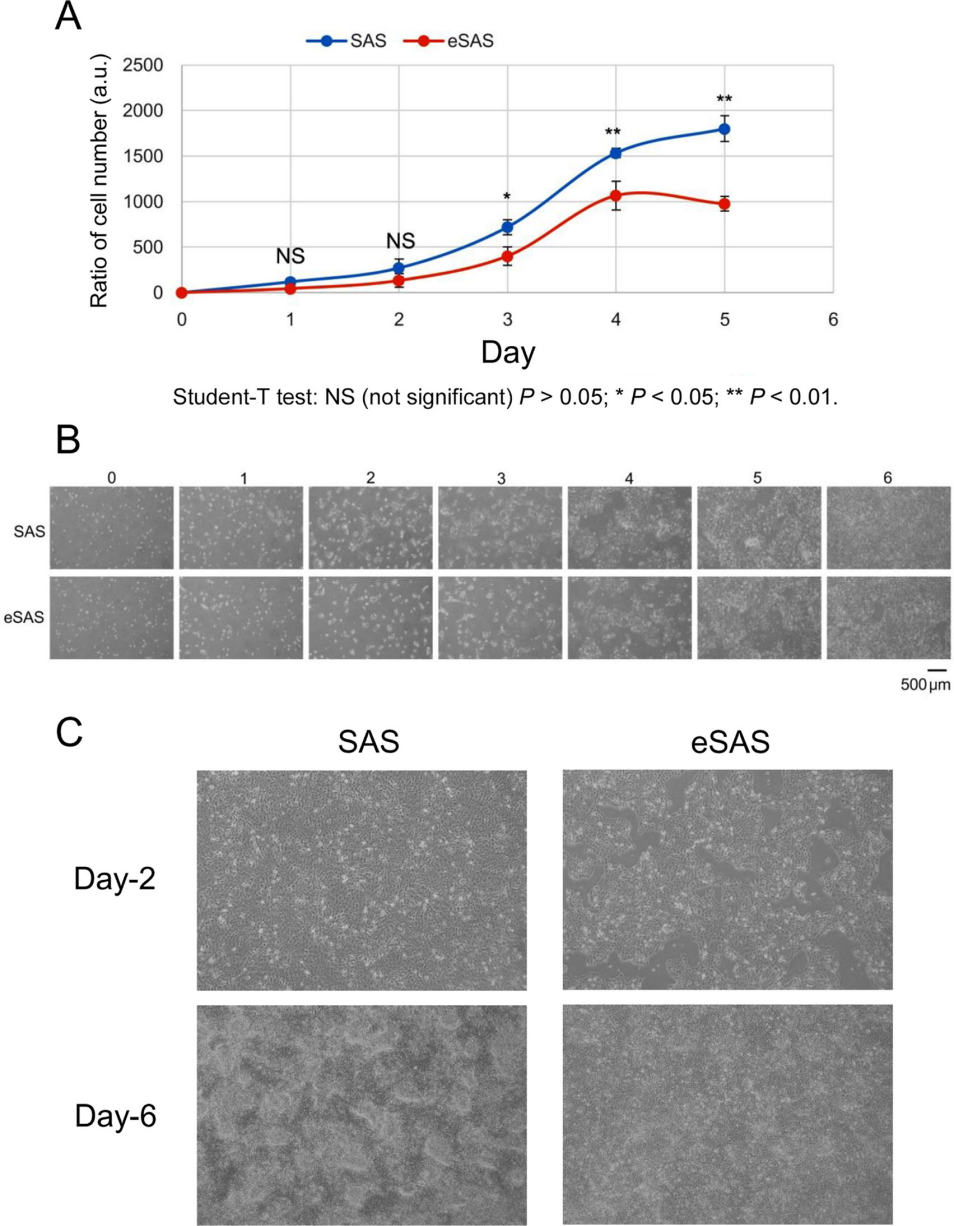
Growth of eSAS cells is slower than that of SAS cells (**A**) Growth of eSAS cells is slower than that of parental SAS cells. The line graph shows the cell number ratio (a.u., arbitrary unit) at the indicated day versus the cell number at day 0, which was defined as the day after one overnight culture of the cells in the culture dish. The ratios of cell numbers at indicated time points were calculated by dividing the cell numbers at the indicated time points by that at day 0. (**B**) Typical microscopic images of SAS and eSAS cells during the time course of incubation to determine cell number ratios. (**C**) Enlarged views of microscopic images of SAS and eSAS cells at days 2 and 6.

To examine whether eSAS cells cultured in standard culture medium and dishes exhibit altered cell growth patterns, we compared cell growth rates between SAS and eSAS cells. We found that the eSAS cells grow significantly slower than SAS cells in standard culture medium and conventional culture plates (Figure 5A). Photographs of cell shape revealed no significant differences between the cell morphologies of SAS and eSAS cells in early logarithmic (Log) growth phase (days 0–3 in Figure 5B). By contrast, when the cells reached the late Log phase, eSAS cells displayed more “piled-up” growth than SAS cells, which exhibited scattered cell growth in standard culture medium and dish (days 4 and 5 in Figure 5B). Enlarged images of eSAS cells at days 2 and 6 showed that these cells grew more homogeneously than SAS cells, which displayed “piled-up” growth in standard culture medium (Figure 5C). These results suggest that we can successfully distinguish eSAS cells from parental SAS cells based on their large spheroid forming ability, slower growth rates, and different growth patterns in late log phase.

### Wound healing, invasion and migration assays

**Figure 6:**
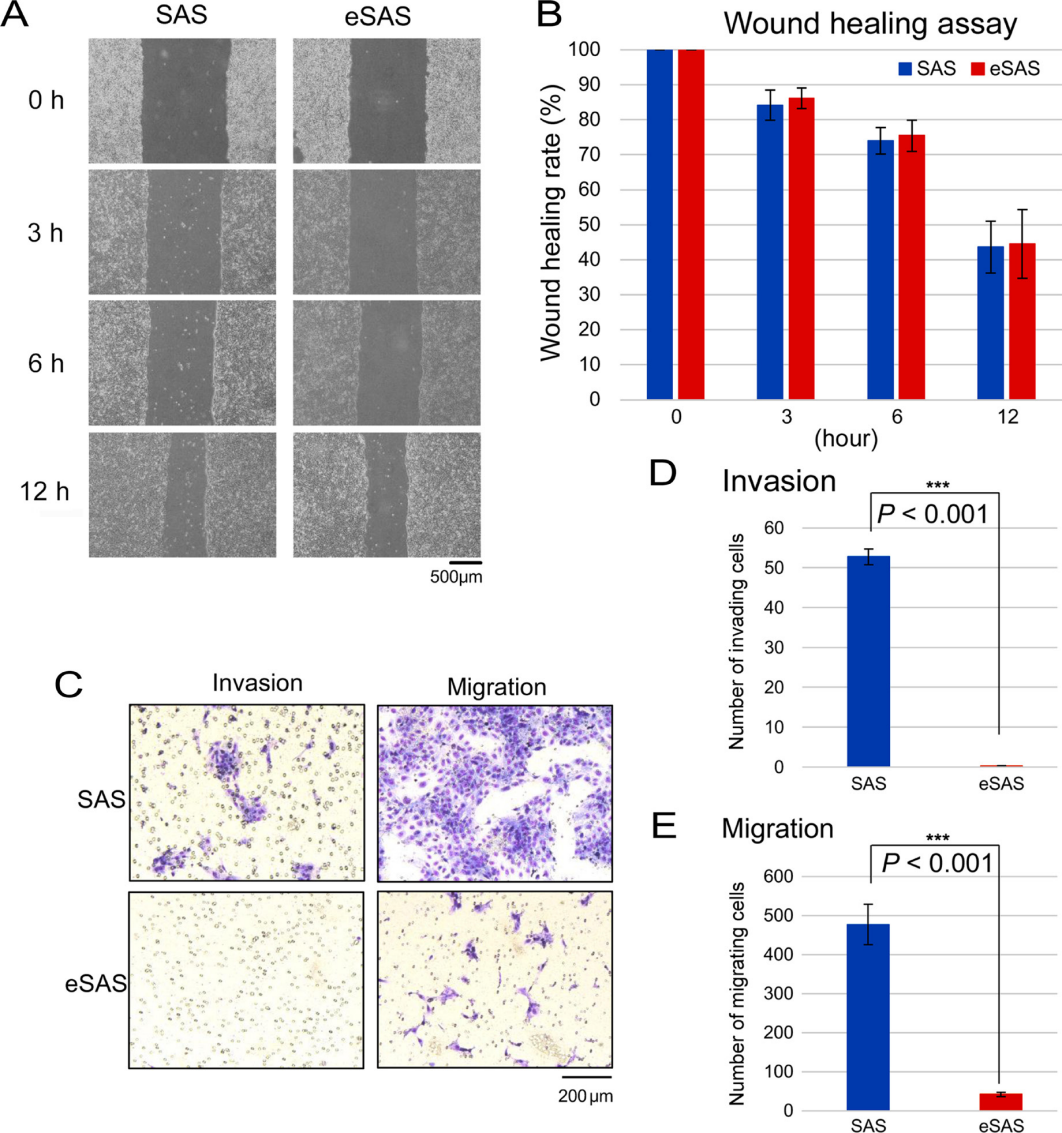
Wound healing, invasion, and migration assays of SAS and eSAS cells (**A**) Typical microscopic images of SAS and eSAS cells at the indicated time points after uniformly scratching away a part of a confluent monolayer of SAS and eSAS cells with a 1 mL pipette tip. (**B**) Bar graph displays the rate at which the width (distance between both edges of the wound) of the scratched area was recovered by cell motility. The ratio of the width at the indicated time points was calculated by dividing each value by the value at time zero (100%). Five points of the wound were appropriately selected and measured. (**C**) Typical image of invading and migrating SAS and eSAS cells on the membrane after the invasion and migration assays for SAS and eSAS cells. Scale bar, 200 μm. (**D**, **E**) Bar graphs represent the number of invading (D) and migrating (E) SAS and eSAS cells on the membrane. The numbers of invading cells and migrating cells were expressed as the average number of cells per microscopic field over five fields and over three fields, respectively.

To examine whether eSAS cells display other distinguishing characteristics, we performed a wound healing assay, which estimates the ability of endothelial cells to initiate migration when a scratch is made in a confluent culture [14]. When the times required to repopulate the scratch made in confluent cellular monolayers of SAS and eSAS cells were compared, we found little difference in the time required for wound healing between the two cell types (Figure 6A and 6B).

We also conducted a cell invasion assay that monitors cellular movement through the extracellular matrix and found that the number of invasive eSAS cells that traversed the matrix was lower than that of the parental SAS cells (Figure 6C and 6D), which suggests that eSAS cells are less invasive than parental SAS cells. Furthermore, the number of migrating eSAS cells was markedly lower than that of the parental SAS cells in the migration assay (Figure 6C and 6E). These results suggest that eSAS cells have a reduced potential for invasion and migration.

### Genome-wide cDNA microarray analyses of gene expression profiles

**Figure 7:**
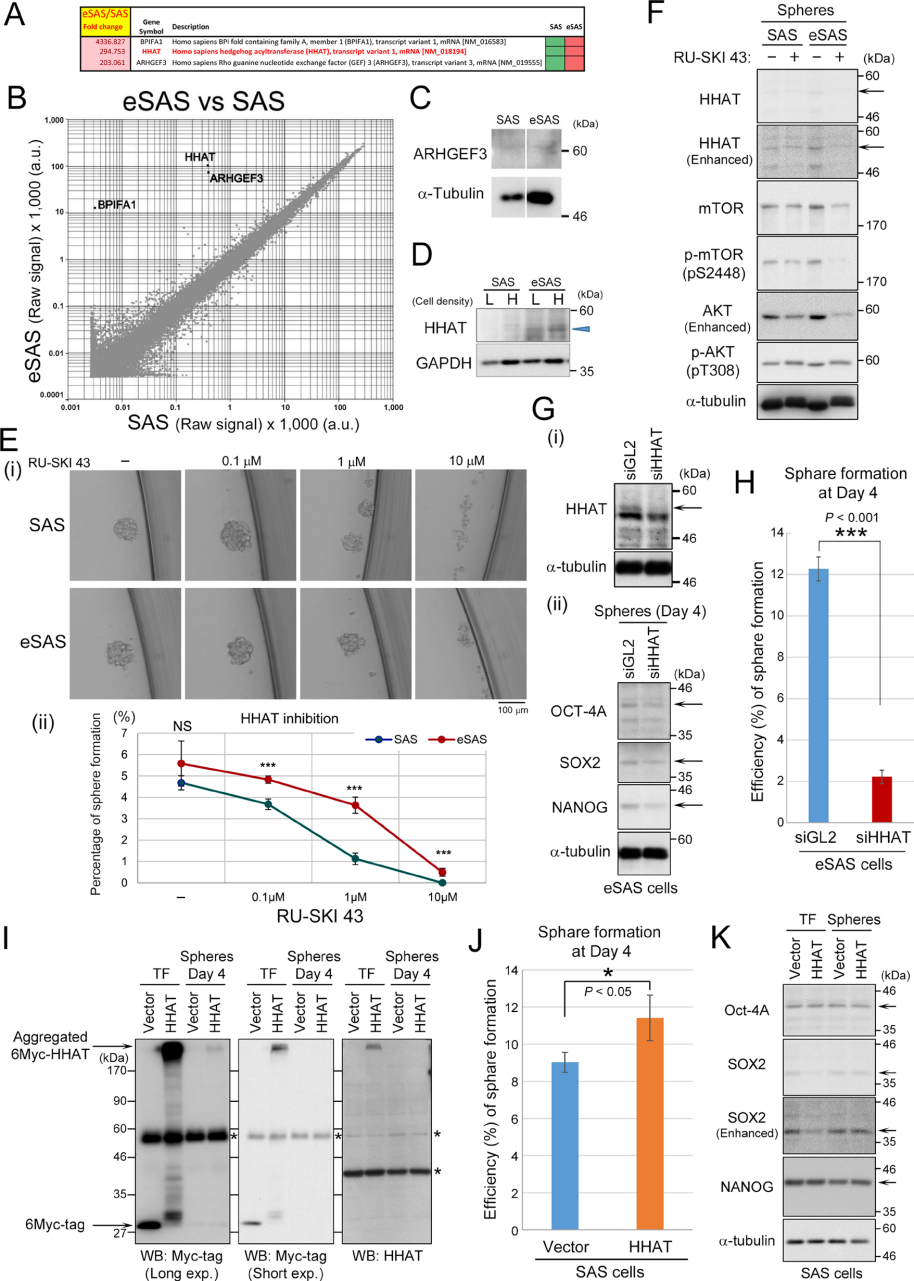
Genome-wide cDNA microarray and Wb analyses of HHAT expression in eSAS cells (**A**) List of fold-changes in BPIFA1, HHATA, and ARHGEF3 mRNA levels, showing that theses gene are conspicuously upregulated in eSAS cells compared with SAS cells. (**B**) Scatter plot of the average expression values of eSAS samples versus SAS samples from DNA microarray analysis, indicating that the mRNA levels of HHATA and ARHGEF3 are differentially expressed in eSAS cells versus SAS cells and that these differences are statistically significant. The mRNA level of the BPIFA1 gene was very low in the SAS group, which resulted in erroneous fold-change values. (**C**) Wb analysis indicating that the expression of ARHGEF3 at the protein level was not significantly different between eSAS and SAS cells. (**D**) Wb analysis demonstrated that HHAT protein levels were higher in eSAS cells than in SAS cells at both low (L) and high (H) cell densities (blue arrowhead). (**E**) Addition of RU-SKI 43 to eSAS and SAS cells under SFM/SFP conditions. Typical microscopic image (i), and line graphs showing the average percentage value for spheroid formation of three independent experiments at each concentration of RU-SKI 43 (ii). (**F**) Wb analysis of the spheres from SAS and eSAS cells with (+) or without (−) 10 μM RU-SKI 43 for 4days using the antibodies against HHAT, mTOR, phosphorylated-mTOR (pS2448), AKT, and phosphorylated-AKT (pS308). Arrows show the bands of HHAT protein. α-tubulin is a loading control. (**G**-i) Wb analysis of the lysates from eSAS cells transfected with siRNA against HHAT (siHHAT) or luciferase (siGL2) as a negative control. Arrow shows the band of HHAT. α-tubulin is a loading control. (G-ii) Wb analysis of the spheres from eSAS cells knocked-down HHAT. Arrows show the band of stem cell markers. α-tubulin is a loading control. (**H**) Bar graphs indicate the percentages of sphere formation for eSAS cells transfected with siHHAT or siGL2 at day 4. Bar graphs were constructed based on results of three independent experiments. (**I**, **K**) SAS cells were transfected with 6Myc-tagged HHAT or 6Myc-vector alone. At 48 hours after transfection (TF), the cells were cultured for 4 days under SFM/SFP condition to generate spheroids. Wb analysis of the lysates from TF or spheres using the antibodies against Myc-tag and HHAT (**I**) or stem cell markers (**K**). Asterisks show non-specific bands. (**J**) Bar graphs indicate the percentages of sphere formation for SAS cells transfected with 6Myc-tagged HHAT or 6Myc-vector alone at day 4. Bar graphs were constructed based on results of three independent experiments.

To examine whether gene expression patterns are different between eSAS and parental SAS cells, we performed genome-wide cDNA microarray analyses. For this purpose, we extracted total mRNA from the SAS and eSAS cells during log growth phase and compared their genome-wide expression levels. We found that three genes were conspicuously upregulated in the eSAS cells but not in SAS cells (Figure 7A and 7B). Bactericidal/permeability-increasing protein fold-containing family member A1 (BPIFA1) encodes a secretory protein called palate, lung, and nasal epithelium clone protein (PLUNC), which inhibits epithelial sodium channels and participates in innate immune responses to bacteria in airways [19]. Because the large fold-change in the expression of this gene was mainly attributed to its very low expression level in SAS cells and because its function is not related to spheroid formation and cancer, we did not subject this protein to further investigation in this study. ARHGEF3 encodes Rho-guanine nucleotide exchange factor (GEF) 3, whose expression level is positively associated with metastasis and more advanced clinical stages of nasopharyngeal carcinoma [20]. However, Wb analysis showed little difference in its protein expression between the SAS and eSAS cells (Figure 7C).

By contrast, hedgehog acyltransferase (HHAT) gene expression is associated with cell growth and is suggested to play a role in cancer by promoting the N-terminal palmitoylation of sonic hedgehog (SHH) [21], which is required for proper SHH signaling involved in the control of cell growth. Wb analysis demonstrated higher HHAT protein levels in eSAS cells than in SAS cells at both low and high cell densities (Figure 7D: also see Figure 3C). Notably, the addition of RU-SKI 43, a potent HHAT inhibitor, reduced the formation and maintenance of spheroids in a concentration-dependent manner under SFM/SFP conditions (Figure 7E-i). In these experiments, the spheroid formation ability of eSAS cells was higher than that of the SAS cells irrespective of the concentration of RU-SKI 43 employed (Figure 7E-ii). To confirm the inhibitory effect of RU-SKI 43 on HHAT protein or its activity, we examined the HHAT protein levels after inhibition (Figure 7F). The HHAT protein levels were slightly decreased in eSAS cells after treatment with RU-SKI 43. Because it has been previously reported that HHAT inhibition by RU-SKI 43 negatively affected Akt and mTOR pathways in pancreatic cancer cells [22], we also assessed the impact of RU-SKI 43 on Akt and mTOR pathways in SAS and eSAS cells. The protein and phosphorylation (activation) levels of Akt and mTOR were decreased after inhibition of HHAT, especially in eSAS cells. These results suggest that RU-SKI 43 actually inhibited HHAT and its downstream pathways in eSAS cells. Moreover, we also confirmed that the specific inhibition of HHAT by siRNA reduced HHAT protein levels in eSAS cells (Figure 7G-i, arrow) and demonstrated that the knockdown of HHAT apparently suppressed the spheroid formation (Figure 7H). Furthermore, the knockdown of HHAT also slightly reduced the protein levels of stem cell markers, such as OCT-4A, SOX2, and NANOG (Figure 7G-ii, top, second, and third panels). Conversely, overexpression of HHAT increased the efficiency of sphere formation in SAS cells, although overexpressed HHAT protein was aggregated and unseparated on SDS-PAGE (it may be due to the property of transmembrane protein of HHAT) (Figure 7I and 7J). Notably, the transient overexpression of HHAT in SAS cells caused downregulation of SOX2 after transfection (Figure 7K, second and third panels, lanes ‘TF’), although HHAT has been reported to be transcriptionally upregulated by SOX2 [23]. These results suggest that HHAT may act in a negative feedback loop to block abnormal overexpression of SOX2. Unexpectedly, however, the expression levels of stem cell markers, including SOX2, OCT-4A, and NANOG, were not changed by overexpressing HHAT in growing spheres (Figure 7K). These results suggest that high HHAT protein expression plays an important role in large-sized spheroid formation (see Discussion).

## DISCUSSION

In the present study, we used an apparatus called Spheroid Catch that yielded cells with increasing spheroid formation ability, namely aSAS, bSAS, cSAS, dSAS, and eSAS cells at the first, second, third, fourth, and fifth selection, respectively (Figures 1A and 2A). Compared to aSAS cells, eSAS cells displayed more spheroid-forming ability (Figure 2C–2H) and a higher resistance to cisplatin in SFM on a non-adherent plate (Figure 3A). Notably, after incubation in standard culture medium on a conventional adherent plate for 10 days (Figure 2B), eSAS cells still showed higher spheroid-forming ability (Figure 2C–2H). The growth rate of eSAS cells in standard culture medium on conventional adherent plates was slower than that of SAS cells (Figure 5A and 5C). Nonetheless, when we performed orthotopic implantation into the tongues of nude mice, the survival rate of mice injected with the eSAS cells was lower than that of mice injected with parental SAS cells (Figure 4A). Histopathological analysis revealed that eSAS cells grew faster and formed larger tumors than SAS cells when implanted into the tongues of nude mice (Figure 4B). Although the precise reason why the mice died earlier is unclear, it is possible that the larger tumor size interfered with eating and the mice died earlier from malnutrition (Figure 4A). Moreover, invasive eSAS cells were observed in the lymph nodes of mice implanted with eSAS cells but not in those implanted with SAS cells (Figure 4C). However, eSAS cells were less invasive and migratory than parental SAS cells in tissue culture plates (Figure 6C–6E). It is possible that growth rate (Figure 5) and invasion (Figure 6) assays using tissue cultured cells do not reflect accurately growth (Figure 4B) and invasiveness *in situ* (Figure 4C). One possible explanation for these seemingly contradictory results is that eSAS cells with higher spheroid-forming ability might have a higher graft-survival rate than SAS cells in the tongue where the rapid blood flow could allow even a small number of spheroid-forming cells to initiate growth and differentiation, thereby promoting engraftment of the cells, tumor formation, and death of the implanted nude mice. Another possibility is that eSAS cells may be specialized cells with the enhanced ‘epithelial’ characteristics or the enhanced cell-cell contact. Although it is technically difficult to prove these conjectures at present, future work employing a new technique will be directed toward clarify this point.

Genome-wide cDNA microarray and Wb analyses demonstrated higher HHAT protein expression in eSAS cells than in SAS cells at both low and high cell densities. It remains to be determined if this result may provide any hint to elucidate our supposition or not. HHAT, a membrane protein that is predominantly located in the endoplasmic reticulum, catalyzes the N-palmitoylation of Hedgehog (HH) proteins, such as sonic HH (SHH), which are secreted from cells and function as morphogens in a concentration-dependent manner [24]. HH proteins play essential roles in embryonic patterning during development and in tumorigenesis. Indeed, total RNA sequencing of mRNA obtained from oncosphere cells after siRNA-mediated knockdown of the PRKKC gene, in combination with Meta pathway analysis, showed that the HHAT gene was strongly associated with stem maintenance in lung squamous cell carcinoma [23]. The PRKKC gene is required for the transformation and expansion of bronchio-alveolar stem cells [25] and is also upregulated in lung cancer cells [23]. Moreover, the survival of colon CSCs was recently reported to be dependent on HHAT-mediated palmitoylation of SHH [26]. These results point to the possibility of using the HHAT as a therapeutic target for the development of anti-cancer drugs, and in particular, against malignant tumors that contain stem-like cancer cells. Enhanced activation of SHH signaling has been linked to the progression of breast and pancreatic cancers [27, 28], and selective HHAT chemical probes [22] and several inhibitors have been developed as potential therapeutic agents [24, 28].

Our results show that Spheroid Catch has potential for the selection of large organoids, suggesting that it could be used in basic and translational research focused on personalized medicine. An organoid is a small-scale organ produced by 3D tissue culture from one or a few cells derived from a tissue in the presence of a specific combination of factors [29], and has potential use as a model system to study human organ development and pathologies including cancer [30]. Efficient selection of large organoids will likely promote the rapid development of techniques for growing organoids. Spheroid Catch may be useful for the isolation of heterologous cancer tissue-originated spheroids (CTOSs) obtained by the dissociation of cancer tissues using mild enzymatic treatments [1, 31]. Because tumor-originated spheroids are considered to include enriched CSCs or cells with stem cell-related characteristics, Spheroid Catch may promote the discovery of CSCs in surgical specimens from cancer patients [1, 31]. Notably, CTOSs form xenograft tumors that retain the features of the parental tumors, suggesting that primary cancer cells from surgical specimens could provide a unique preclinical model for personalized medicine [31]. Moreover, Spheroid Catch could be used for the selection of large human organoids obtained via induced pluripotent stem cell (iPSC) technology, which could help promote the practical use of iPSC technology and make iPSC-based programs even more influential in precision medicine [32].

## MATERIALS AND METHODS

### Cell culture

A human oral squamous cell carcinoma cell line derived from a tongue tumor, SAS, was obtained from the Human Science Resource Cell Bank (Osaka, Japan). SAS cells were cultured in Dulbecco's modified Eagle's medium (DMEM, Sigma, St. Louis, MO) supplemented with 10% fetal bovine serum (FBS, Hyclone, Logan, UT), 100 U/mL penicillin, and 100 μg/mL streptomycin, and incubated at 37°C and 5% CO_2_.

### Protocol for Spheroid Catch

SAS cells were incubated in SFM on a cell culture plate with a low-adhesion surface (EZ-BindShut^®^II of AGC TECHNO GLASS, Japan) at 37°C in a 5% CO_2_ incubator for several days (Figure 1A-i). SFM (~10 mL) contained 5 mL DMEM, 5 mL Ham's F-12K (Kaighn's) Medium, 200 ng epidermal growth factor, 200 ng basic fibroblast growth factor, and 0.2 mL B-27 (×50). A Spheroid Catch, which was obtained from Fukae Kasei Co. Ltd. (Kobe Japan), was set up in a collection tube (e.g., 50 mL centrifuge tube; T2318, Sigma-Aldrich). SAS cells were collected and transferred gently from the culture plate to the Spheroid Catch set up in a collection tube (Figure 1A-i~iii). The plate was rinsed with PBS to collect the spheroids that were tightly attached to the culture plate, and the spheroids were transferred from the culture plate to the Spheroid Catch. After gravity filtration, the selection of large spheroids was increased by removing small-sized spheroids by centrifuging the collection tube containing Spheroid Catch at 190 × *g* for 5 s at room temperature. (Figure 1A-iv). The mesh was detached by creating a small hole at the bottom of the Spheroid Catch with a needle or a tip of forceps (Figure 1A-v) and transferred (Figure 1A-vi) to a conventional culture plate (diameter, 3.5 cm) containing 1 mL accumax (Innovative Cell Technologies) and incubated at 37°C in a 5% CO_2_ incubator for 7 min to disperse the aggregated cells (Figure 1A-vii). Then, the cells were mixed with 2 mL standard culture medium (DMEM containing 10% FBS) and the mixture was transferred to a 15-mL centrifuge tube (T1818, Sigma-Aldrich). The cells were collected by centrifugation (190 × *g*) for 5 min at room temperature (Figure 1A-viii). The supernatant was discarded and the solution including precipitated cells at the bottom (~1 mL) was transferred to a 1.5 mL microfuge tube (Figure 1A-iX). The cells were disaggregated by repeated suction and release using a 1 mL syringe equipped with a 25 G needle (Figure 1A-x), which completely disperses the spheroids into single cells. The number of single cells was counted. In selection step #2 (Figure 1A-xi), cells from selection step #1 were resuspended in SFM and plated at density of ~400/cm^2^ in an EZ-BindShut^®^II (e.g., 3,100 cells per a 10-cm diameter plate). This selection step may be repeated five times (Figure 1A-xii~xv). To establish a cell line, a single cell was selected by resuspending ~100 cells in 10 mL of SFM, transferring 0.1 mL of SFM into each well of a 96-well plate, and incubating for more than a week at 37°C in a 5% CO_2_ incubator until spheroids were observed in each well.

### Single colony isolation and assay for spheroid formation efficiency

Approximately ten cells, counted by an automated cell counter, Countess II (Thermo Fisher Scientific), were seeded into each well of a 96-well EZ-BindShut^®^II plate. Following an overnight incubation step, the number of surviving cells was counted under a microscope. Next, at the indicated time point, the number of putative spheroids was counted and the efficiency of sphere formation was calculated by dividing the number of the surviving cells by the number of seeded cells. The numbers of seeded cells were measured by counting live cells after 16 h incubation under a microscope. At day 4 (Figure 2G), a colony harboring more than ten cells was defined as a spheroid. At day 7 (Figure 2F), a colony displaying an irregular-sized globular shape and covered with putative extracellular matrix was also defined as a spheroid. To confirm that the increased spheroid-forming ability is a stably-acquired characteristic, the captured spheroids of aSAS or eSAS were enzymatically and physically dispersed, and then the dispersed cells derived from each spheroid were cultured in a conventional adherent condition for 10 days, followed by reformation of spheroids under SFM/SFP conditions (Figure 2B–2D).

### Wound healing assay

SAS and eSAS cells were grown to confluence and washed with PBS(−) that is a phosphate buffered saline without calcium and magnesium ions. Next, the cell surface was uniformly scratched with a 1 mL pipette tip. After removing the detached cells by washing with PBS(−), the adherent cells were cultured in the culture medium at 37°C and 5% CO_2_ for the indicated time. Cell motility was assessed by measuring the distance between both edges of the scratch. Five points in the scratch were randomly selected and measured.

### Cell invasion assay and migration assay

A two-layer Transwell chamber (Corning BioCoat Matrigel invasion chamber, 8.0 μm pore size) was used to perform cell invasion assays according to the manufacturer's instructions (Thermo Fisher Scientific, Waltham, MA). Briefly, we seeded the cells in the upper chamber containing 2 mL of DMEM without serum at a density of 5.0 × 10^5^ cells/mL, and added only DMEM with 10% FBS to the lower chamber. After incubation at 37°C with 5% CO_2_ for 72 h, we removed the cells in the upper chamber by scrubbing with a cotton-tipped swab. Next, we stained the cells on the lower surface of the membrane using a Diff-Quik kit (Sysmex, Kobe, Japan).

We performed a cell migration assay using a Transwell chamber (Falcon cell culture inserts; Thermo Fisher Scientific) that had a two-layer structure. We counted the number of the cells that invaded or migrated through the membrane on microscope photographs of the membrane. The numbers of invading cells and migrating cells were expressed as the average number of cells counted under the microscope (Leica DM2000).

### Cell growth assay

Cells were seeded at a density of 0.5 × 10^5^ per well in a 6-well plate, and cultured at 37°C and 5% CO_2_. The cells were counted using the Countess automated cell counter (Invitrogen) every day for six days. We defined the day after one overnight culture of the cells as day 0. The ratios of cell numbers at indicated time points were calculated by dividing the cell numbers at the indicated time points by the cell number at day 0.

### Murine tongue tumor model

SAS and eSAS cells (5 × 10^5^ cells in 50 μL of serum-free DMEM) were injected into the tongues of 6-week-old female nude mice (BALB/c Slc-*nu*/*nu*; Japan SLC Inc., Shizuoka, Japan) using a syringe with a 26 G needle. After injection, primary tumor formation was identified by visual observation, and the survival rates and body weights were measured. Four tongue tumors were excised from four mice sacrificed on day 8 and were examined pathologically. The tissue section (3 μm) samples were prepared by The Research Foundation for Microbial Diseases of Osaka University [BIKEN] after fixation in a 10% formalin neutral buffer solution (Wako Pure Chemical Industries, Ltd., Osaka, Japan).

### Plasmids, siRNAs, and transfection

Human HHAT variant 1 (NM_018194) was generated by PCR using KOD-plus polymerase (TOYOBO, Japan) and cloned into mammalian expression vector pCMV6myc at the *Asc*I and *Not*I sites. The amplified sequences were confirmed by DNA sequencing. The plasmids were purified by the QIAfilter Plasmid Midi Kit (QIAGEN). SAS cells were transfected with pCMV6myc-HHAT or vector alone using Lopofectamine and PLUS reagent (Invitrogen). At 48 hours after transfection, the cells were cultured for 4 days under SFM/SFP conditions to form spheroids. Sequences of siRNA duplexes were as follows: siHHAT, 5′-UUAAUCAGGUAUGUGUACAUUCCAGUGdGdA-3′; and siGL2 (firefly luciferase), 5′-CGUACGCGGAAUACUUCGAdTdT-3′. For the knockdown of HHAT, eSAS cells were transfected with siHHAT or siGL2 using Lipofectamine 2000 (Invitrogen). At 24 hours after transfection, the cells were cultured for 4 days under SFM/SFP conditions to form spheroids.

### Antibodies

Monoclonal antibodies raised against the following proteins were purchased from the indicated commercial sources: α-tubulin (Sigma-Aldrich, #T5168), Myc-tag (MBL, #M047-3), and GAPDH (MBL, #M171-3). Polyclonal antibodies raised against the following proteins were purchased from the indicated commercial sources: ARHGEF3 (Abcam, #ab154263), HHAT (ABGENT, #AP5503a for Figures 3C, 7D, 7F, and 7I; and Sigma, #SAB2105163 for Figure 7G-i), OCT-4A (Cell Signaling, #2840), SOX2 (Cell Signaling, #3579), NANOG (Cell Signaling, #4903), mTOR (Cell Signaling, #2983), mTOR-pS2448 (Cell Signaling, #5536), AKT (Cell Signaling, #75692), and AKT-pS308 (Cell Signaling, #13038).

### Western blot analysis

Cell extracts were prepared using modified TNE250 lysis buffer (10 mM Tris-HCl [pH 8.0], 250 mM NaCl, 1 mM EDTA, 0.25% NP-40, 1 mM dithiothreitol, and 2 mM benzamidine) or RIPA lysis buffer (20 mM Tris-HCl [pH 7.5], 150 mM NaCl, 1% Triton X-100, 1% sodium deoxycholate, 0.1% SDS) supplemented with 100 μg/mL PMSF, 1 μg/mL aprotinin, 10 μg/mL leupeptin, 1 μg/mL pepstatin A, 1 mM NaF, 1 mM Na_3_VO_4_, 10 mM β-glycerophosphate, and 100 nM okadaic acid at 4°C for 30 min [33]. After centrifugation, cleared lysates were denatured with SDS-sample buffer. The proteins were separated by SDS-PAGE and transferred to PVDF membranes, followed by western blot analysis with the indicated primary antibodies at 1:100 (HHAT) and 1:500 (ARHGEF3, mTOR, mTOR-pS2448, AKT, AKT-pT308, OCT-4A, SOX2, and NANOG) dilutions in TBST (20 mM Tris-HCl [pH 7.5], 150 mM NaCl, and 0.05% Tween 20) with 5% non-fat milk or bovine serum albumin (BSA) after blocking. The HHAT antibody (ABGENT) or another HHAT antibody (Sigma) were diluted in TBST with 5% non-fat milk or 5% BSA, respectively. The membranes were probed with HRP-conjugated secondary antibodies, washed seven times for 5 min each in TBST at room temperature, and visualized using the Western Lightning Plus ECL (PerkinElmer, Waltham, MA).

### DNA microarray

DNA microarray analysis was performed as described previously [10] at the DNA-chip Development Center for Infectious Diseases, RIMD, Osaka University. The detailed microarray data have been deposited in the Gene Expression Omnibus (GEO; www.ncbi.nlm.nih.gov/geo) database (accession number GSE106207).

### Statistical analysis

Statistical analysis was performed using Excel. Error bars for the data on cell growth and invasion/migration assays represent the standard deviation from the mean. *P*-values were calculated using the Student's *t*-test. Survival analysis of nude mice was evaluated by the Kaplan-Meier method and the results obtained were compared with the log-rank test (Cochran-Mantel-Haenszel). ^*^P < 0.05, ^**^P < 0.01, ^***^P < 0.001.
